# Hyperoxia and Lungs: What We Have Learned From Animal Models

**DOI:** 10.3389/fmed.2021.606678

**Published:** 2021-03-09

**Authors:** Luciano Amarelle, Lucía Quintela, Javier Hurtado, Leonel Malacrida

**Affiliations:** ^1^Department of Pathophysiology, Hospital de Clínicas, School of Medicine, Universidad de la República, Montevideo, Uruguay; ^2^Advanced Bioimaging Unit, Institut Pasteur Montevideo and Universidad de la República, Montevideo, Uruguay

**Keywords:** hyperoxia, animal models, translational science, lung injury, hyperoxia acute lung injury

## Abstract

Although oxygen (O_2_) is essential for aerobic life, it can also be an important source of cellular damage. Supra-physiological levels of O_2_ determine toxicity due to exacerbated reactive oxygen species (ROS) production, impairing the homeostatic balance of several cellular processes. Furthermore, injured cells activate inflammation cascades, amplifying the tissue damage. The lung is the first (but not the only) organ affected by this condition. Critically ill patients are often exposed to several insults, such as mechanical ventilation, infections, hypo-perfusion, systemic inflammation, and drug toxicity. In this scenario, it is not easy to dissect the effect of oxygen toxicity. Translational investigations with animal models are essential to explore injuring stimuli in controlled experimental conditions, and are milestones in understanding pathological mechanisms and developing therapeutic strategies. Animal models can resemble what happens in critical care or anesthesia patients under mechanical ventilation and hyperoxia, but are also critical to explore the effect of O_2_ on lung development and the role of hyperoxic damage on bronchopulmonary dysplasia. Here, we set out to review the hyperoxia effects on lung pathology, contributing to the field by describing and analyzing animal experimentation's main aspects and its implications on human lung diseases.

## Introduction

Aerobic respiration is a vital process in mammalian cells in which adequate oxygen (O_2_) delivery is essential. Nevertheless, supra-normal levels of O_2_ can damage cellular constituents and thus trigger cell injury and death (1, 2). The lung is the first organ affected by hyperoxia, but increasing evidence indicates that high blood concentration of O_2_ (hyperoxemia) can also determine harmful systemic consequences (3). Acute lung injury is featured by diffuse alveolar damage with interstitial and alveolar edema due to increased alveolar-capillary permeability to liquid, proteins and inflammatory cells (4). Hyperoxia is a frequent iatrogenic consequence of oxygen therapy, which can induce pulmonary damage and maximize mechanical ventilation associated acute lung injury, leading to severe consequences on gas exchange and respiratory mechanics (2, 5, 6). Moreover, in the last few years clinical reports have shown an increased mortality in patients with hyperoxia in intensive care units (7, 8). The lungs of critically ill patients are often exposed to several insults, such as mechanical stress, infections, hypo-perfusion, systemic inflammation and drug toxicity. In these scenarios it is not easy to clearly dissect the effect of oxygen toxicity. Translational investigations with animal models are essential to exploring injuring stimuli in controlled experimental conditions, and are milestones in the understanding of pathological mechanisms and the development therapeutic strategies. Here, we set out to review the animal models used to investigate hyperoxia and its effects on different pathological situations such as acute lung injury, chronic respiratory diseases and impairment of pulmonary development, among others.

## Methods

An electronic search of Pubmed was made to identify the eligible studies, involving those published until 2020, combining the following keywords: hyperoxia; lung injury; animal models. All articles and cross-referenced studies were screened for appropriate information and reviewed by the authors. Inclusion criteria included original experimental and review articles. Publications not written in English or Spanish were excluded.

## Oxygen Toxicity Mechanism

The homeostatic balance of cellular processes can be disrupted when exposed to supra-physiological concentrations of O_2_, due to exacerbated reactive oxygen species (ROS). Production of ROS is directly proportional to tissue O_2_ concentration, as they are intermediate metabolites produced during aerobic metabolism. Mitochondrial ROS generation begins with superoxide anion (O_2_^**•−**^) the main sources of which are complex I and II electron transfer. Further reactions lead to hydrogen peroxide (H_2_O_2_), hydroxyl radical (OH^**•−**^) and peroxynitrite anion (ONOO^−^) formation, all of which are highly reactive molecules able to damage intracellular components, proteins, lipids and nucleic acids. Antioxidant cell defense mechanisms, including superoxide dismutase, catalase and peroxidase enzymes, and non-enzymatic compounds (low molecular weight scavengers, proteins, glutathione), are overwhelmed when mitochondrial ROS production increases in the presence of hyperoxia (9–11). Injured cells activate inflammation cascades and cytokines such as interleukin-1, interleukin-6, and interleukin-8, which have an important role on amplifying the tissue damage by attracting and activating neutrophils, macrophages and other inflammatory cells, causing increased vascular permeability and secondary ROS production. After secondary injury, both endothelial and epithelial cells are damaged, therefore alveolar-capillary barrier integrity is lost, leading to interstitial edema (2, 12, 13).

## Animal Models

Since O_2_ was described by Lavoisier on 18th century, pioneer investigators observed that animals breathing under high O_2_ atmosphere suffered severe lung inflammation and died within a few hours. In an article published in 1849, Lorrain Smith reported that hyperbaric hyperoxia on mice, rats, guinea pigs and birds, resulted in convulsions and lung congestion leading to death (14). During the 20th century, experiments on different species (rabbits, cats, dogs, monkeys, mice and rats), reported a lethal toxicity of oxygen. In general, these animals developed progressive respiratory distress and died from respiratory failure in between 3 and 6 days (12). The injury level and the exposure time needed to induce death vary notably among animal species. Mortality studies show that a fraction of 0.7 inspired O_2_ (FIO_2_) was the upper limit beyond which toxic effects are clinically relevant (15, 16).

### Large Animal Models of Hyperoxic Acute Lung Injury (HALI)

Primates were used to model human disease since they are evolutionarily closer to humans than other mammals. HALI was observed in baboons, with progressive pulmonary damage characterized by destruction of endothelial and alveolar type I cells, hypertrophy of type II cells, interstitial edema and neutrophil accumulation (17). In terms of recovery, monkeys who survived a hyperoxic environment and who were allowed to reach full recovery showed normal lung histology suggesting that sub-lethal HALI has a reversible pattern (18). Oxygen toxicity was also proposed as a second hit injury. Studies in baboons revealed a synergistic effect of hyperoxia and bacterial pneumonia on lung injury (19), however, experiments with hyperoxia exposure after oleic acid infusion suggested that previous lung injury does not change the response to hyperoxia (20). Paradoxically, rabbits pre-treated with oleic acid were found to develop a delayed oxygen toxicity and prolonged survival, compared with a non-pre-treated hyperoxic group (21, 22). Furthermore, a potential benefit of hyperoxia was proposed by Milstein et al., who observed in rabbits that hyperoxia induces changes in microcirculatory flow in a reversible manner with a slight impact on macro-hemodynamic parameters (23).

### Large Animal Models of Bronchopulmonary Dysplasia (BPD)

The effect of a high oxygen dose during the early stages of life is known to have deleterious effects on lung development. Experimentation on animal models has been essential to understanding the role of hyperoxia and the underlying mechanisms of BPD, a chronic lung disease in which the development of the respiratory system is severely affected. Rabbit models helped to elucidate some important features of the BPD in preterm born infants. The transcriptome analysis on preterm pups shows that the main pathways altered by hyperoxia are related with reactive oxygen species production, inflammation, and lung and vascular development (24). Other reports observed that the gestational age and the FiO_2_ are determinant factors for BPD, inducing characteristic histological changes and decreasing survival rates (25). Moreover, Jiménez et al. have reported that preterm rabbits exposed to up to seven days of hyperoxia show early structural changes in the lung vasculature and alveoli development compromised with severe consequences on respiratory physiology (26). Nonetheless, short term noninvasive ventilation with 100% O_2_ as a strategy for preserving spontaneous breathing was shown to be beneficial in preterm rabbits (27). Additionally, therapeutic studies on large animal models have contributed to proposed therapeutic strategies, such as the pharmacological effect of statins on preventing the arterial remodeling and BPD induced by hyperoxia in a rabbit model (28) and the protective effect of the proton-pump inhibitor Omeprazole in a rabbit model of neonatal hyperoxic lung injury by inducing Cytochrome P4501A1 (CYP1A1) activation (29). Moreover, larger preterm animals such as lambs, baboons, and pigs (30–33) have been studied to model human neonatal disease, in which hyperoxia has a key role.

### Small Animal Models of Hyperoxic Acute Lung Injury (HALI)

Following the 3Rs (Replacement, Reduction, and Refinement) recommendations in the bioethics principles (34), and considering that large animal models are often costly and time-consuming, small animals have been used for alternative models for biological research.

#### Rat Models

Rats breathing 24 h in a 100% oxygen atmosphere showed an increase of stress response gene HO-1 (heme oxygenase-1) in lung tissue homogenates (35), and at 48 h the pulmonary surfactant was altered, leading to a decrease in lung compliance after 60 h of hyperoxia (36). Moreover, in a short exposure of 90 min to hyperoxia, inflammatory cells and biomarkers of oxidative stress were increased and histology revealed lung injury in a dose dependent manner (37). Furthermore, experiments in rats revealed that lung injury after 60 h of hyperoxia could be paradoxically prevented by the free radical nitric oxide when it was added to the hyperoxic gas mixture (38).

#### Mice Models

At present, mice models are the most used to study hyperoxia due to the wide availability of transgenic strains, essentials to explore the pathophysiology of HALI. The susceptibility to hyperoxia was shown to vary between mice and rats and within different mice strains. More than 40 years ago, Tierney et al. observed that mice exposed to an enriched oxygen atmosphere for several days have an increased mortality rate when compared to rats (39). Recent research demonstrated that the susceptibility to oxygen toxicity in mice depends on three main factors, (1) sex: the adult C57BL/6 WT male mice had more lung injury and inflammatory edema after hyperoxia exposure (40–42). Also, neonatal male C57BL/6 WT are more susceptible to oxygen toxicity in terms of inflammatory response and impairment in lung development (43–45). Nevertheless, a different sex susceptibility can be observed in other strains. In genetic analysis, Prows et al. showed that in adult mice resulted from the cross of sensitive (C57BL/6) and resistant (29X1/SvJ) progenitor strains, females have increased susceptibility to hyperoxia compared with males (46, 47). Considering the strain and the sex differences in terms of susceptibility is imperative when analyzing the effects of hyperoxic damage. (2) age: the survival rate is inversely correlated with age, and (3) strain: C3H/HeJ and 129X1/SvJ mice are resistant to hyperoxic damage, whereas C57BL/6J background confers a consistent sensitivity to the strain (47–49). In terms of genetic research, Prows et al. demonstrated that the quantitative trait loci *Shali-1* and *Shali-2* have strong effects on survival times after hyperoxia (46, 50). Experimental hyperoxia on transgenic mice have helped us to understand the pathophysiology of HALI and to elucidate mediators, signaling pathways and cell death mechanisms involved. Some important processes of oxygen toxicity include cytokine (IL-3) and grow factors (TGFß, VEGF, Ang2) release, activation of transmembrane receptor (P2X7) and intracellular pathways including inflammasome, kinases cascades, and oxygen reactive species processes. Many transgenic mice strains have been used to explore hyperoxia, showing different susceptibility patterns, in Table 1 the main reports are listed, describing gene alteration, background strain, sex, age, method of exposure to hyperoxia, outcomes measured and susceptibility pattern (Table 1).

**Table 1:** Transgenic mice and susceptibility to hyperoxia.

**Transgenic mice**	**Background strain**	**Sex**	**Age**	**Phenotype/Alterations**	**Hyperoxia exposure**	**Outcome measured**	**Effect**	**References**
**Cytokines**								
CC10–IL-11	C57BL/6	UNS	UNS	Overexpression of IL-11 in Clara cells of the lung	100% O_2_ chamber	ALI markers. Oxidative stress. Survival rate	Protection	(51)
CC10–IL-6	C57BL/6	UNS	UNS	Overexpression of IL-6 in Clara cells of the lung	100% O_2_ chamber	ALI markers. Oxidative stress. Survival rate	Protection	(52)
IL-3 KO	C57BL/6	Male	7–9 weeks	Deficiency of the pro inflammatory cytokine interleukin (IL)-3	100% O_2_ chamber	ALI markers	Protection	(53)
**Growth factors**								
Ang2 KO	C57BL/6	UNS	4–6 weeks	Deficiency of Angiopoietin 2, a regulator of angiogenesis and vascular homeostasis	100% O_2_ chamber	ALI markers. Apoptosis. Survival rate	Protection	(54)
TGFβ1R2 KO	C57BL/6	UNS	1 day	Deficiency of the type II transforming growth factor beta 1 receptor (TGFβR2). TGFβ is a secretory cytokine regulator of proliferation, differentiation and apoptosis. It is involved in many pathological processes.	100% O_2_ chamber (7 days)	Alveolarization. ALI markers. Apoptosis. Survival rate	Protection	(55)
VEGF-D KO	C57BL/6	Male	7–10 weeks	Deficiency of the vascular endothelial growth factor D, an angiogenic and lymphangiogenic protein	95% O_2_ chamber	ALI markers	Protection	(56)
**Receptors**								
P2X7 KO	C57BL/6	Female and male	6 weeks	Deficiency of the purinergic receptor P2X7, a membrane receptor involved in innate and adaptive immune responses.	100% O_2_ chamber	ALI markers. Survival rate	Protection	(57)
**Cell signaling and pathways**								
Src^+/−^	C57BL/6	UNS (54)	6–8 weeks (54)	Deficiency of Src (heterozygotes), a tyrosine kinase, which regulate cell processes such as proliferation, migration, apoptosis	100% O_2_ mechanical ventilation	ALI markers. Apoptosis	Protection	(58, 59)
		Male (55)	2–3 month (55)					
Nlrp3 KO	C57BL/6	Female and male (56)	3 days (56)	Deficiency of the Nucleotide-binding domain and leucine-rich repeat PYD-containing protein 3, an essential component of the inflammasome.	85–100% O_2_ chamber	Alveolarization. ALI markers. Apoptosis	Protection	(60–62)
		Female (57)	8–14 weeks (57)					
		UNS (58)	7–9 weeks (58)					
ASK1 KO	C57BL/6	UNS	7–9 weeks	Deficiency of the apoptosis signal-regulating kinase 1 (ASK1), a member of the mitogen-activated protein kinase (MAP3K) family, involved in cell response to stress.	100% O_2_ chamber	ALI markers. Apoptosis	Protection	(63)
SphK1 KO	C57BL/6	Female	6 weeks	Deficiency of sphingosine kinase (SphK)1, which is involved in ROS production by NOX activation	95% O_2_ chamber	ALI markers. Oxidative stress	Protection	(64)
**Other transporters, enzymes and metabolic factors**								
db/db	C57BL/6	Male	6–8 weeks	Leptin resistance Mice are obese, hyperphagic and hyperglycemic	100% O_2_ chamber	ALI markers	Protection	(65)
SP-D KO	C57BL/6	Male	7–8 weeks	Deficiency of the surfactant protein D	80% O_2_ chamber	ALI markers. Oxidative stress. Survival rate	Protection	(66)
Periostin KO	B6/129	UNS	2–3 days	Deficiency of the protein Periostin, a non-structural extracellular matrix- associated molecule	75% O_2_ chamber (14 days)	Alveolarization	Protection	(67)
	(Control: C57BL/6 WT)							
sEH KO	C57BL/6	Male	7–10 weeks	Deficiency of the soluble epoxide hydrolase, an enzime with a role on lipid metabolism	100% O_2_ chamber	ALI markers. Survival rate	Protection	(68, 69)
Tgm2 KO	C57BL/6	Female and male	1 day	Deficiency of the transglutaminase (TGM)-2, important for extracellular matrix (ECM) structure and function	85% O_2_ chamber (14 days)	Alveolarization	Protection	(70)
Cyp1b1 KO	C57BL/6	Female and male	8–10 weeks	Deficiency of the cytochrome P450 enzyme CYP1B1	>95% O_2_ chamber	ALI markers. Oxidative stress	Protection	(71)
slc7a2 KO	C57BL/6	Female and male	8–12 weeks	Deficiency of the cationic amino acid transporter (CAT)-2, by which L-arginine is transported to participate in NO production	>95% O_2_ chamber	ALI markers. Lung mechanics. Apoptosis	Protection	(72)
**Cytokines**								
IL-13 KO	C57BL/6	UNS	4–6 weeks	Deficiency of IL-13	100% O_2_ chamber	ALI markers. Apoptosis. Survival rate	Susceptibility	(73)
**Growth factors**								
Fgf10^+/−^	C57BL/6	Female and male	0 day	Deficiency of Fibroblast growth factor 10 (FGF10) (heterozygotes), a protein with a key role in embryonic lung development	85% O_2_ chamber (8 days)	Alveolarization	Susceptibility	(74)
**Receptors**								
CD44 KO	C57BL/6	Male	10–12 weeks	Deficiency of the transmembrane adhesion molecule CD44	>95% O_2_ chamber	ALI markers. Survival rate	Susceptibility	(75)
TLR4 KO	C57BL/6	UNS	6–10 weeks	Deficiency of toll-like receptor 4	100% O_2_ chamber	ALI markers. Apoptosis. Survival rate	Susceptibility	(76)
TREK KO	C57BL/6	Female and male	9–12 weeks	Deficiency of the two-pore domain potassium (K2P) channel. Mice lack TREK-1, TREK-2, and TRAAK isoforms (triple KO).	95% O_2_ chamber and mechanical ventilation	ALI markers. Lung mechanics. Apoptosis	Susceptibility	(77)
**Cell signaling and pathways**								
NRF2 KO	ICR/Sv129 (74)	Female and male (74)	6–8 weeks (74)	Deficiency of the transcription factors NRF2, a regulator of antioxidant genes	95–99% O_2_ chamber	ALI markers. Apoptosis	Susceptibility	(78, 79)
	C57BL/6 (75)	UNS (75)	8 weeks (75)					
NOS2 KO	C57BL/6	UNS	4–6 weeks and 1 day	Deficiency of nitric oxide (NO) synthase 2, an enzyme which generates NO	100% O_2_ chamber	ALI markers. Apoptosis. Survival rate	Susceptibility	(80)
**Other transporters, enzymes and metabolic factors**								
Bfl-1/A1 KO	C57BL/6	UNS	6–8 weeks	Deficiency of the anti-apoptotic protein Bfl-1/A1, a Bcl-2 family member	100% O_2_ chamber	ALI markers. Apoptosis. Survival rate	Susceptibility	(81)
TIMP-3 KO	C57BL/6	Male	9–12 weeks	Deficiency of the tissue inhibitors of metalloproteinases. Determines chronic and progressive lung air space enlargement	>90% O_2_ chamber	Lung mechanics. ALI markers	Susceptibility	(82)
BRP-39 KO	C57BL/6	Female and male	4–6 weeks	Deficiency of Breast regression protein −39, a chitinase-like protein	100% O_2_ chamber	ALI markers. Apoptosis. Survival rate	Susceptibility	(83)
Ddit3 KO	C57BL/6	Female	UNS	Deficiency of CCAAT enhancer-binding protein homologous protein (CHOP), a pro-apoptotic transcription factor	>95% O_2_ chamber	ALI markers. Survival rate	Susceptibility	(84)
AhRd	C57BL/6	UNS	0 day	Aryl hydrocarbon dysfunctional B6.D2N-Ahrd/J. AhRd is a regulator of detoxification enzymes. This transgenic mice have decreased affinity of the receptor AhRd for its ligand	85% O_2_ chamber (14 days)	Alveolarization	Susceptibility	(85)
Hsp70 KO	C57BL/6	UNS	6–10 weeks	Deficiency of heat shock proteins 70s	100% O_2_ chamber	ALI markers. Apoptosis. Survival rate	Susceptibility	(76)
Cyp1a1 KO	C57BL/6	UNS	8–10 weeks	Deficiency of the cytochrome P450 enzyme CYP1A1 and CYP1A1	95% O_2_ chamber	ALI markers. Oxidative stress	Susceptibility	(86, 87)
apoE KO	C57BL/6	Male	8–15 weeks	Deficiency of apolipoprotein E (apoE), involved in the lipid transport. Mice have hypercholesterolemia, premature atherosclerosis and impaired inflammatory response.	100% O_2_ mechanical ventilation	ALI markers	Susceptibility	(88)
dnTrx-Tg	C57BL/6	UNS	UNS	Decreased levels of functional thioredoxin (Trx), an antioxidant protein.	>90% O_2_ chamber	ALI markers. Survival rate	Susceptibility	(89)
LysM-Cre/Foxm1 KO	C57BL/6	UNS	0 day	Selective deficiency of Foxm1 in myeloid-derived inflammatory cells.	85% O_2_ chamber (21 days)	Alveolarization	Susceptibility	(90)
ogg-1 KO	C57BL/6	UNS	UNS	Deficiency of the 8-oxoguanine- DNA glycosylase (OGG)-1, an enzyme involved in DNA repair	95 % O_2_ chamber	ALI markers	Susceptibility	(91)
Akap1 KO	C57BL/6	UNS	7–9 weeks	Deficiency of the mitochondrial A-kinase anchoring protein (Akap), a regulator of the mitochondrial function.	100% O_2_ chamber	ALI markers	Susceptibility	(92)

*Uns, Unspecified in the original article; Ali, Acute lung injury; PN, Post-natal*.

#### Hyperoxia and Second Hit Models of HALI in Small Rodents

In addition to the described for large animals (20–22), small rodents have also been used to study the role of hyperoxic insult superimposed on a preexisting damage, enhancing its effect (a second hit). Rats exposed continuously to 100% oxygen are more susceptible to toxic acute lung injury with a marked increase in mortality rate (39). Also, hamsters breathing on 70% O_2_ atmosphere for 72 h after bleomycin instillation showed more pulmonary fibrosis and higher mortality (15). Hakkinen et al. observed similar effect on rats with bleomycin and cyclophosphamide induced lung injury followed by exposition to 80% oxygen for 6 days (93). Moreover, rats have been essential for research on lung mechanical stress and its effects on respiratory physiology. The characterization of the mechanical ventilation induced lung injury (VILI) determined a paradigm shift in the management of critically ill patients. Furthermore, the hyperoxic acute lung injury was proposed to be an additional mechanism on VILI. High tidal volume (Vt) ventilation of 20 ml/kg plus high FiO_2_ for 2 h was shown to cause significantly more pulmonary edema and neutrophil migration on Sprague-Dawley rats. The mechanical stress can induce chemoattractant MIP-2 (macrophage inflammatory protein-2), which could have a role on this inflammatory response to VILI and HALI (94). Additionally, it was observed in a rat model that the synergistic effect of VILI and HALI impaired alveolar type II cell adhesion due to changes in adhesion proteins by RhoA signaling activation (95). In terms of susceptibility, aged Wistar rats had a more severe lung damage and diaphragmatic dysfunction in a model of short term mechanical ventilation and hyperoxia when compared with adult rats (96). Furthermore, the enhanced effect of hyperoxia on VILI was also widely studied in mice as is discussed in the acute exposure section of this review. Second hit impact of hyperoxia was additionally demonstrated in a rat model of fulminant sepsis, in which increasing FiO_2_ directly affected mortality rates in a dose dependent manner (97). Experiments in adult mice also observed increased inflammatory injury in with 100% hyperoxia for 48 h after acute lung injury induced by intra-tracheal administration of lipopolysaccharide (LPS) and staphylococcal enterotoxin B (98). These models are relevant for translational analysis, considering that most critically ill patients exposed to high doses of oxygen by mechanical ventilation have some pulmonary or systemic additional disease, resulting in a multi factorial insult to the lungs in which hyperoxia has an important role.

### Small Animal Models of Bronchopulmonary Dysplasia (BPD)

Modeling the effects of hyperoxia on the neonatal in mice and rats has many advantages, e.g., they have relatively short gestation times and short lifetimes and are less expensive than larger animals. However, perhaps the most important benefit is that mice and rats are delivered at term in the saccular stage of lung development, like preterm infants that develop BPD. Development of the lung is typically described to occur over five stages: embryonic, pseudo-glandular, canalicular, saccular, and alveolar stages in overlapping periods (99–101). Term human babies (38 week-old) are born in the alveolar phase, whereas preterms between 24–36 weeks are born in the saccular stage. On the other hand, the saccular phase of rats and mice starts on the embryonic day 20 and 17, respectively, and both end in the postnatal day 5, where the alveolar phase starts. Comparison of developmental stages among different animal species can be found in recent state-of-the-art reviews (30, 32, 101, 102). Hyperoxia has been widely used to model BPD as it determines an impaired alveolarization, increased collagen deposition, and interstitial thickness, associated with changes in lung function. Cell proliferation is inhibited, whereas inflammatory cell activation and its products are exacerbated. Proinflammatory cytokines, such as TNF-α, IL-6, and IL-1, play a key role in hyperoxic oxidative lung damage by altering essential growth factors and thus have been proposed as therapeutic targets (103–105). Prolonged exposition to high FiO_2_ in sealed chambers for several days is the usual model to induce BPD and evaluate the effect of hyperoxia on the impairment of lung development. The dose needed to develop BPD in murine models are frequently very high (FiO2 >0.8), which does not precisely correlate with the dose used usually in patients. Interestingly, lower FiO_2_ was demonstrated to cause structural alteration in lung tissue, and a dose dependent effect was observed (106–108). Moreover, Yee et al. reported a model closer to the clinical course in human preterm neonates using concentrations from 0.4 to 1 for PN days 1–4, followed by 8 weeks of room air recovery (107). The duration of exposure is also a critical factor in the effect of hyperoxia in lung development. In many studies, continue exposure from PN day 0–28 was used to reproduce histopathological changes similar to BPD (103, 109, 110). Nevertheless, a shorter exposure also induces a marked impairment in lung tissue development (111–114). Consistently, *in vitro* studies have demonstrated robust changes in cellular metabolism with alteration of oxidative phosphorylation triggered by only 4 h of hyperoxia (114).

#### Effects of Hyperoxia on Pulmonary Vasculature on Neonatal Models

Hyperoxia is known to alter growth factor signaling, extracellular matrix (ECM) assembly, cell proliferation, apoptosis, and vascular development (100, 115). Experiments in animals have shown that hyperoxia impairs angiogenesis by downregulating essential growth factors such as vascular endothelial growth factor (VEGF) (104, 116–120). Additionally, the role of factors such as hypoxia-inducible factor (117, 121, 122) has been studied in animal models and helped to identify potential therapeutic targets. Transforming growth factor-β (TGF-β) plays a pivotal role during lung development and angiogenesis by regulating endothelial cell growth, differentiation and migration, and ECM production. Recent findings on mice models of hyperoxia reported the contribution of two endoglin isoforms (L- and S-endoglin) to TGF-β downstream signaling and its role on angiogenesis and BPD development (123). Moreover, the vascular consequences of hyperoxia exposure during lung development can cause long term alterations that persist into adulthood. Studies in rats demonstrated that hyperoxia alters the reactivity in pulmonary arteries (124), leading to an impairment of the contractile properties of the right ventricle (125). Pulmonary hypertension signs such as arterial thickness and right ventricle hypertrophy were observed in mice with post-natal hyperoxia, among other systemic vascular structural effects (126–128).

#### Hyperoxia and Second Hit Models of BDP in Small Rodents

Hyperoxic insult has also been studied as a second hit factor on BPD. The combination of prenatal infections or hypoxia with post-natal noxious stimuli such as mechanical ventilation and hyperoxia are known to be key pathogenic factors. Using a mice model, Gortner et al. combined prenatal grow restriction by hypoxia with post-natal hyperoxia (129, 130) to induce BPD. Interestingly, when hypoxia in short episodes occurs after hyperoxia, the structural alteration is exacerbated (129). Perinatal inflammation induced by LPS, followed by prolonged exposure to hyperoxia produces BPD and was used as a model to study anti-inflammatory therapeutic strategies (131, 132).

## Discussion

### Chronic Exposure

As a mechanistic approach, experimental animals breathing in a high oxygen atmosphere in a chronic scenario has been widely used. The main method for chronic hyperoxic exposition consists in rooming the animals in closed and sealed chambers with an oxygen enriched atmosphere for several hours or days. This model has been used in many reports with mice and rats, assessing different features of oxygen toxicity. Thereby, exposing rodents to 72 h of a pure oxygen atmosphere in a chamber has been shown to induce lung edema and alveolar leakage with increased oxidative stress markers and recruitment of inflammatory cells and mediators, such as IL1-ß, TNF α and IL-6. Moreover, prolonged exposure causes lung mechanics alterations, increasing airway resistance and diminishing lung compliance; also histological evidence of injury has been consistently observed (53, 57, 72, 77, 92). The extended exposure in a plastic chamber requires an inexpensive system which could be hand crafted, additionally, it is a technically simple and high fidelity reproducible model. It is a suitable method to characterize different phases of acute lung injury and also to observe persistent structural, biochemical and inflammatory changes that can be translated into human diseases such as chronic obstructive pulmonary disease (COPD) with permanent oxygen dependency. Nevertheless, it does not simulate the effect of an acute high fraction of inspiratory oxygen that is frequently delivered to critically ill patients under mechanical ventilation. For this purpose, short exposure models (with and without mechanical ventilation) have been reported and are reviewed in the following section.

### Acute Exposure

The exposure to supra-normal concentration of oxygen in an acute scenario usually involves mechanical ventilation (MV). MV entails profound changes on respiratory physiology by applying positive pressure to the lungs and subjecting the parenchyma to abnormal stretching stress, which, if excessive, could lead to ventilator-induced lung injury (VILI), with structural alteration, impaired gas exchange and activation of the inflammatory cascades (133). Hyperoxia added to MV with high tidal volumes (Vt) establishes a scenario with high risk of acute lung injury and systemic inflammation. Animal models of acute hyperoxia are helpful to evaluate the harmful effects of oxygen and MV, resembling what happens in critical care or anesthesia patients. Hence, rodent models have been used to characterize HALI plus VILI lung injury. The ventilatory pattern with large Vt (20 ml/k) and 100% oxygen was observed to induce more pulmonary edema and inflammatory infiltration than the same Vt without hyperoxia in a rat model (94). Pre-exposure to a high oxygen environment was also shown to aggravate lung injury produced by mechanical ventilation in adult mice (134). Also, the potential anti-inflammatory effect of pluripotent stem cells was proposed to ameliorate hyperoxia-augmented VILI through the inhibition of Src-dependent signaling pathway (59). Interestingly, Wagner et al. observed that 4 h of low Vt mechanical ventilation with 100% O_2_ did not increase lung injury after blunt chest trauma and cigarette smoking in a mouse model, but paradoxically, decreased nitrosative stress (135). Furthermore, hyperoxia without mechanical ventilation was seen to be harmful in acute experimental conditions, namely, mice with 2 h-exposition to 60% O_2_ showed pulmonary genotoxicity that could be prevented by the halogenated volatile anesthetic Isofluorane by decreasing superoxide anion generation (136). Moreover, in a rat model Nagato et al. observed that 90 min of hyperoxia itself determines lung injury with typical histological changes, immune cells infiltration and excessive oxidative stress (37). All those findings taken together suggest that short term hyperoxia - which is often tolerated in perioperative and severe ill patients - could be as harmful as long term hyperoxia, and should be avoided.

### Modeling the Disease

Critically ill adults and newborn babies are often exposed to several insults, including oxygen toxicity. Translational investigation with animal models is essential to explore injuring stimuli in controlled experimental conditions. Nevertheless, some aspects of animal biology differ markedly from human functional mechanisms. Moreover, the models often do not entirely resemble what happens in human pathology and can give an oversimplified explanation of the mechanisms. Many animal models lack the complexity of multifactorial insults, the interaction of mechanisms, and the individual differences that converge in human disease. To address this point, the second hit injury models previously described have been proposed to study the hyperoxic effect in a scenario closer to human disease. Large animal models can be anatomically similar to humans, appropriate to test some therapeutic strategies (i.e., Mechanical ventilation, non-invasive support), and more accessible to technical instrumentation, but are expensive, time demanding, and offer fewer reagent and transgenic models compared with rodents. Models are not good or bad; the point is to choose a model that can test our scientific hypothesis. Animal models of hyperoxia are fundamental to understanding lung diseases and the development of therapeutic strategies.

In summary, hyperoxia is a frequent harmful consequence of oxygen therapy, leading to lung injury and exacerbating the effect of other insults. Translational investigation with animal models is essential to study the toxicity of oxygen in a complete system and explore the mechanisms involved, safety levels, and potential therapeutic targets. In this narrative review, we contribute to the research field by describing and analyzing the main aspects of animal models of hyperoxia and its implications on human pathology.

## Author Contributions

LA and LQ carried out the database search and wrote the manuscript. LA, LM, and JH reviewed the manuscript. All authors contributed to the article and approved the submitted version.

## Conflict of Interest

The authors declare that the research was conducted in the absence of any commercial or financial relationships that could be construed as a potential conflict of interest.
